# Prediction of Extreme Response of Nonlinear Oscillators Subjected to Random Loading Using the Path Integral Solution Technique

**DOI:** 10.6028/jres.099.044

**Published:** 1994

**Authors:** Arvid Naess

**Affiliations:** Faculty of Civil Engineering, The Norwegian Institute of Technology, Rich. Birkelands vci la, N-7034 Trondheim, Norway

**Keywords:** extreme response, nonlinear oscillators, path integral solution, random loading

## Abstract

This paper studies the applicability of the path integral solution technique for estimating extreme response of nonlinear dynamic oscillators whose equations of motion can be modelled by the use of Itô stochastic differential equations. The state vector process associated with such a model is generally a diffusion process, and the probability density function of the state vector thus satisfies the Fokker-Planck-Kolmogorov equation. It is shown that the path integral solution technique combined with an appropriate numerical scheme constitutes a powerful method for solving the Fokker-Planck Kolmogorov equation with natural boundary conditions. With the calculated probability density function of the state vector in hand, one can proceed to calculate the required quantities for estimating extreme response. The proposed method distinguishes itself by remarkably high accuracy and numerical robustness. These features are highlighted by application to example studies of nonlinear oscillators excited by white noise.

## 1. Introduction

An important element in the safety assessment of many engineering systems, is the task of estimating the probability of extreme events that may jeopardize the structure in some specified sense. Very often, this problem can be formulated as finding the probability that some time varying random quantity does not exceed a specified capacity level during a given time period. Stated this way, the problem typically reduces to a study of the extreme values of a stochastic process originating as the response of a system subjected to some stochastic loading process.

In this paper the focus will be on the problem of estimating the extreme response of nonlinear dynamic systems subjected to random forcing processes. In recent years the methods of time domain Monte Carlo simulations, see, e.g., Refs. [1–5], have received considerable attention as a tool for estimating response statistics. These methods are versatile and attractive in the sense that nonlinearities can be easily dealt with. The main drawback at present is the large CPU times needed for accurate prediction of extreme responses. Even if this issue seems to become less of an obstacle every year, portending perhaps that such methods may dominate practical estimation of response statistics of nonlinear systems in the not too distant future, it will still be desirable to have available alternative methods of calculating the response statistics, both simplified and more elaborate. Here we shall explore a method based on the theory of Markov diffusion processes. The justification for using this theory is related to the fact that the response of nonlinear dynamic systems to broad band random excitation can very often be accurately described by applying the theory of multidimensional Markov processes. By this, the extensive theory of Markov diffusion processes can be brought to bear on these problems. In particular, it can be shown that the probability law of response quantities can be derived by solving a partial differential equation, viz., the Fokker-Planck (-Kolmogorov) (FPK) equation, see Refs. [6,7]. In most cases of practical interest, this equation has to be solved numerically.

In the next section we shall describe a method for solving the FPK equation that is based on a formal solution of the same equation. This solution is obtained by invoking the fact that a Markov diffusion process locally looks like a Brownian motion. By using the Markov property, the global solution can then be constructed by linking the local solutions, which are known explicitly. The obtained solution is generally known as a path integral solution (PIS). The reader is referred to Ref. [7] for a further discussion. One of the first efforts to exploit the PIS method explicitly in developing numerical solution algorithms is described in Ref. [8]. Subsequently, other authors have also used the PIS approach to solve various random vibration problems, cf. Refs. [9–13].

Before embarking on a description of the PIS method, it is expedient to briefly show how the obtained solutions are used in an extreme value analysis. Assuming that the response quantity of interest is a scalar (real) stationary stochastic process, *Z*(*t*) say, the PIS method typically provides a numerical estimate of the joint probability density function (PDF) *fzz*(•,•) of *Z*(*t*) and Ż(*t*)=dZ(*t*)/d*r*. It is now assumed that the mean level upcrossing rate $\nu_{z}^{+}\left(•\right)$ of Z(*t*) can be calculated from Rice’s formula as follows

$$\nu_{Z}^{+}\left(z\right)=\int_{0}^{\infty }yfzz\left(z,y\right)dy.$$ (1)

Adopting the assumption that upcrossing of high levels are statistically independent events, which leads to Poisson distributed crossings, it follows that an asymptotic approximation of the probability distribution function of the extreme value of the process Z(*t*) during a time *T*, denoted by *M*(*T*) (=sup{Z(*t*);O≤*t*≤*T*}). is given by

$$Prob\left\{M\left(T\right)\leq z\right\}=\exp\left\{-\nu_{z}^{+}\left(z\right)T\right\}\left(T\to \infty \right).$$ (2)

The accuracy of Eq. (2) depends to a large extent on the effective bandwidth of the response process Z(*t*). Decreasing bandwidth leads eventually to a significant clumping effect of large response peaks, invalidating the assumption of statistically independent upcrossing of high levels. Methods that aim at correcting for this effect have been proposed for Gaussian (Refs. [14, 15]) and non-Gaussian (Ref. [16]) processes. However, this point will not be pursued any further here. We shall assume that Eq. (2) provides an acceptable approximation. Hence, the central parameter to be determined is the upcrossing frequency $\nu_{z}^{+}\left(•\right)$, which Ls easily calculated once the joint PDF *f_zz_*(•,•) of *Z*(*t*) and Ż(*t*)=dZ(*t*)/d*t* has been made available. In the next section it is shown how this PDF can be calculated for the response of a wide range of nonlinear oscillators subjected to white noise or filtered white noise loading.

## 2. The Path Integral Solution

The path integral solution (PIS) method is suitable for calculating the joint probability density function (PDF) of a vector process *X*(*t*)=[*X*_l_(*t*)….*X_n_*(*t*)]*^T^* (T-transposi-tion) satisfying a stochastic differential equation of the following form, cf. Ref. [6].

dX(t)=m[X(t)]dt+Q[X(t)]dW(t). (3)

Here m(•)=[*m*_l_(•),….,*m_n_*(•)]*^τ^, m_j_*(•) denotes a real function of *n* real variables. Q(•)=(*q*_ij_(•)) denotes an n×m-matrix where each *q_ij_*(•) is a real function of *n* real variables. W(*t*)=[*W*_l_(*t*),…,*W_m_*(*t*)]*^τ^* where *W*_j_(*t*), *j*=I, *…,m* are standard, real Brownian motion processes, which are mutually independent, sec e.g., Refs. [6,7], That is, *E*[*W_j_*(*t*)]=0 and

$$E\left[dW_{i}\left(t\right)dW_{j}\left(l+\tau \right)\right]=\delta_{ij}\delta_{ij+\tau }d\tau ,ij=1,\ldots ,m.$$ (4)

where δ*_xy_*=l for *x=y*, δ*_xy_*=0 for *x≠y.* Equation (4) is a short-hand notation for the relation $E\left[\iint h\left(s,t\right)dW_{i}\left(s\right)dW_{i}\left(t\right)\right]=\delta_{ij}\int h\left(t,t\right)dt$, where *h*(•,•) is a non-random function.

Equation (3) is interpreted here as an Itô stochastic differential equation (SDE). Since it is often relevant to consider Eq. (3) as being obtained as a limit of equations with band limited noise processes, it may happen that *m*(•) should contain correction terms to ensure a consistent limiting solution, cf. Ref. [6]. It is assumed here that this consideration has already been made, and that Eq. (3) has the final form to be used subsequently.

It is demonstrated in Ref. [6] that the solution *X*(*t*) to Eq. (3) is a Markov vector process. Its transition probability density function (TPD), *p*(*x,t | x′,t*′), is defined by the equation

$$Prob\left\{X\left(t\right)\in A|X\left(t\prime \right)=x\prime \right\}=\int \underset{A}{•••}\int p\left(x,t|x\prime ,t\prime \right)dx,$$ (5)

where *A* ⊆ R*^n^* is some event, *x*, *x*′ ∈= R*^n^*, d*x=dx,…dx_n_.* Provided that *m*(•) and *Q*(•) satisfy certain regularity conditions, see Rcf. [6], it can be proved that the TPD *p*(*x,r | x′,t*′) (*t*≥*t*′≥0) is the solution of a partial differential equation of the form

$$\begin{array}{c} \frac{\partial }{\partial t}p\left(x,t|x\prime ,t\prime \right)=-\sum_{i-1}^{\pi }\frac{\partial }{\partial x_{i}}\left[m_{i}\left(x\right)p\left(x,t|x\prime ,t\prime \right)\right]\\ +\frac{1}{2}\sum_{i-1}^{\pi }\sum_{j-1}^{\pi }\frac{\partial^{2}}{\partial x_{i}\partial x_{j}}\left[g_{\mathrm{ij}}\left(x\right)p\left(x,t|x\prime ,t\prime \right)\right], \end{array}$$ (6)

where $G\left(x\right)=\left(g_{ij}\left(x\right)\right)=Q\left(x\right)Q{\left(x\right)}^{T}=\left(\sum_{k-1}^{m}q_{\mathrm{rk}}q_{jk}\right)$, and with initial condition *p*(*x,t′ | x′,t′*)=δ(*x−x′*). G(•) will be called the diffusion matrix and Eq. (6) will be referred to as the Fokker-Planck-Kolmogorov (FPK) equation. Since clearly *Prob*{*X*(*t*)∈R*^n^|X*(*t′*)=*x′*}= 1 the TPD satisfies the following normalization condition

$$\int \underset{R^{n}}{•••}\int p\left(x,t|x\prime ,t\prime \right)dx=1.$$ (7)

Let *f*(*x*,*t*) denote the PDF of the random vector *X*(*t*). If *f*(*x*, *t*′)=*w*(*x*) for some initial PDF *w*(*x*), then it is recognized from Eq. (6) and the relation

$$f\left(x,t\right)=\int \underset{R^{n}}{•••}\int p\left(x,t|x\prime ,t\prime \right)w\left(x\prime \right)dx\prime$$ (8)

that *f*(*x*, *t*) itself is a solution of Eq. (6) satisfying the initial condition *f*(*x, t*′)=*w*(*x*).

In this paper we shall be interested primarily in stationary solutions *f*,(*x*) to Eq. (6), that is

$$f_{s}\left(x\right)=\underset{t\to \infty }{\lim}f\left(x,t\right)=\underset{t\to \infty }{\lim}p\left(x,t|x\prime ,t\prime \right)$$ (9)

provided they exist. Even when both limits exist, it is clear that lim *f*(*x*, *t*) provides the faster convergence when the initial condition *f*(*x*, *t*′)*≈f*,(*x*). This comment is relevant to the numerical implementation of the PIS method, and will be discussed below.

To obtain the PIS appropriate for the dynamic systems studied in this paper, it is necessary to be more specific on the structure of the matrix function Q(•). In particular, it will be assumed that the first *r* rows of *Q*(•) are zero, that is

$$q_{ij}\left(•\right)\equiv 0\text{for}\;i=1,\ldots ,r;j=1,\ldots ,m\left(r<n\right)$$ (10)

and that *q_ij_*(•)*≠*0 for at least one *j* for every *i*=*r*+l,…,*n.* This implies that the diffusion matrix *G*(•) assumes the form

$$G\left(•\right)=\left[\begin{array}{cc} O & O\\ O & G\left(•\right) \end{array}\right].$$ (11)

*O* denote appropriate zero-matrices and $\tilde{G}\left(•\right)$ denotes an (*n*−*r*)×(*n*×*r*)-matrix function with elements $g_{ij}\left(•\right),ij=r+1\ldots .,n,\tilde{G}\left(•\right)$ will be called the reduced diffusion matrix. Equation (6) can now be rewritten as

$$\begin{array}{l} \frac{\partial }{\partial t}p\left(x,t|x\prime ,t\prime \right)=-\sum_{i-1}^{\pi }\frac{\partial }{\partial xi}\left[m,\left(x\right)p\left(x,t|x\prime ,t\prime \right)\right]\\ +\frac{1}{2}\sum_{i-r+1}^{\pi }\sum_{j-r+1}^{\pi }\frac{\partial^{2}}{\partial x_{i}\partial x_{j}}\left[g_{ij}\left(x)p(x,t|x\prime ,t\prime \right)\right]. \end{array}$$ (12)

Proceeding in a manner similar to the derivations in Ref. [7], it can be shown that the TPD for small values of *τ*(=*t*−*t*′) is given by the following expression, which is correct up to terms of order *τ*^2^

$$\begin{array}{c} p\left(x,t+\tau |x^{\prime },t\right)-\left\{\prod_{i-1}^{r}\delta \left(x_{i}-x_{j}^{r}-m_{i}\left(x^{\prime }\right)\tau \right)\right\}\\ •\left(2\pi \tau \right)-\frac{n-r}{2}|\tilde{G}\left(x^{\prime }\right)|-\frac{1}{2}•\exp\{-\frac{1}{2\tau }\sum_{i-r+1}^{\pi }\sum_{j-r+1}^{\pi }\\ \left(x_{i}-x_{j}^{r}-m_{i}\left(x\prime \right)\tau \right){\left[\tilde{G}{\left(x^{\prime }\right)}^{1}\right]}_{irj-r}\left(x_{j}-{x_{j}}^{r}-m_{j}\left(x^{\prime }\right)\tau \right)\}. \end{array}$$ (13)

where $\left|\tilde{G}\right|$ denotes the determinant of the reduced diffusion matrix $\tilde{G}$, assumed to be positive definite. This implies that $\left|\tilde{G}\right|>0,[{\tilde{G}}^{-1}{]}_{\mathrm{ij}}$, denotes the element in position *i j* of the inverse matrix of $\tilde{G}$. As shown in Ref. [7], the expression given by Eq. (13) is not unique, but seems to be well suited for our purpose.

Having obtained an explicit expression for the TPD for a short time step, one can now invoke the Markov property. This allows a TPD over a time interval of arbitrary length to be expressed in terms of a product of short-time TPDs. By dividing a given time interval (*t*′,*t*) into *N* small time intervals of length τ=(*t−t′*)/*N*, it is found that (*t_j_=t′+jτ, t=t^N^, x*=*x*^(N)^, *t′=t*_0_, *x′=x*^(0)^)

$$p\left(x,t|x\prime ,t\prime \right)=\int_{R^{n(N-1)}}•••\int \prod_{j-1}^{N}p\left(x^{\left(j\right)},t_{j}|x^{\left(j-1\right)},t_{j-1}\right)dx^{\left(1\right)}•••dx^{\left(N-1\right)}.$$ (14)

Similarly, with an initial PDF *f*(*x′,t′*)*=w*(*x′*), the PDF *f*(*x,t*) will be given by

$$f\left(x,t\right)=\int \underset{{R^{n}}^{N}}{•••}\int \prod_{j-1}^{N}p\left(x^{\left(j\right)},t_{j}|x^{\left(j-1\right)},t_{j-1}\right)w\left(x^{\left(0\right)}\right)dx^{\left(0\right)}•••dx^{\left(N-1\right)}.$$ (15)

Hence, by combining Eq. (13) with Eqs. (14) or (15), a formal (approximate) solution of the FPK equation can be written. Equations (14) and (15), which arc often referred to as PIS, constitute the core of the numerical solution procedure to be described subsequently. It is realized that a numerical solution according to this method, automatically provides the evolution in time of the (conditional) PDF of the Markov process *X*(*t*) from given start conditions in terms of an initial density *f*(*x′, t′*)*=w*(*x′*), including the degenerate case *f*(*x′, t′*)=δ(*x′*−*x*_0_), for some starting point *x*_0_. It is also worth noting how the PIS relates to the physics of the dynamic model, which is expressed through the coefficients *m_j_*(•) and *q_ij_*(•), cf. Eq. (3). The evolution in time of the PDF as expressed by the PIS, is seen to be directly determined by these coefficients in an explicit manner. This fact is a very important advantage of the PIS method, and reveals its fundamental physical significance.

## 3. Numerical Implementation

In the numerical implementation, the PIS is obtained by an iteration process based on the Chapman-Kolmogorov equation expressed as

$$\begin{array}{c} p\left(x^{\left(j\right)},t_{j}|x\prime ,t\prime \right)=\int \underset{R^{n}}{•••}\int p\left(x^{\left(j\right)},t_{j}|x^{\left(j-1\right)},t_{j-1}\right)p(x^{\left(j-1\right)},\\ t_{j-1}|x\prime ,t\prime )dx^{\left(j-1\right)}. \end{array}$$ (16)

The discretization of state space for the numerical solution makes it appropriate to employ an interpolation and smoothing procedure to increase the numerical efficiency. It was found that application of cubic B-splines, as detailed in Ref. [17], offered the desired accuracy and smoothness for the type of problems considered in this paper. This procedure was used as follows. At each time step *t_j_*_‒1_,→*t_j_*, *p*(*x*^(^*^j^*^‒1)^,*t_j_*_‒1_|*x*′,*t*′) is represented as a cubic B-spline series in the following manner

$$\begin{array}{c} p\left(x^{\left(j-1\right)},t_{j}|x\prime ,t\prime \right)-\sum_{ki-1}^{M_{1}}•••\sum_{k_{n}-1}^{M_{n}}\Gamma^{\left(j-1\right)}\left(k_{1},•••,k_{n}\right)\\ \overset{n}{\underset{j-1}{⊗}}B_{ki}\left(x^{\left(j-1\right)}\right), \end{array}$$ (17)

where *M_j_*=number of grid points for the i’th state variable ${\left\{{⊗}_{i-1}^{n}B_{k},\left(•\right)\right\}}_{k_{j-1}}^{M_{i}}$ is a tensor product basis of cubic B-splines and ${\left\{\Gamma^{\left(j-1\right)}\left(k_{1},•••,k_{n}\right)\right\}}_{k_{i}-1}^{M_{r}}$ is the set of interpolation coefficients associated with time *r_j_*_−1_. It is assumed that each set ${\left\{B_{k_{i}}\left(•\right)\right\}}_{k_{i}-1}^{M_{1}},i=I,•••,n$, is a basis of cubic B-splines associated with the knot sequence determined by the grid points for the i’th variable *x,.* The tensor product B-spline is defined by

$$\overset{n}{\underset{i-1}{⊗}}B_{k_{1}}\left(x\right)=\prod_{i-1}^{n}B_{k_{1}}\left(x_{1}\right).$$ (18)

The representation of *p*(*x*^(^*^j^*^‒1)^,*t_j_*_‒1_|*x*′,*t*′) by B-splincs makes it possible to retain high numerical accuracy even with a fairly coarse basic grid if *p*(*x*^(^*^j^*^‒1)^,*t_j_*_‒1_|*x*′,*t*′) is not too singular. By substituting from Eq. (17) into Eq. (16), Eq. (19) is obtained

$$\begin{array}{c} p\left(x\left(j\right),t_{j}|x\prime ,t\prime \right)=\sum_{k_{1}-1}^{M_{1}}•••\sum_{k_{n}-1}^{M_{n}}\Gamma^{\left(j-1\right)}\left(k_{1},•••,k_{n}\right)\\ \int \underset{R^{n}}{•••}\int p\left(x^{\left(j\right)},t_{j}|x^{\left(j-1\right)},t_{j-1}\right)\overset{n}{\underset{j-1}{⊗}}B_{k_{i}}\left(x^{\left(j-1\right)}\right)dx^{\left(j-1\right)}. \end{array}$$ (19)

It is seen from Eq. (13) that since *m_j_*(•) and *g_ij_*(*•*) are not functions of time *t*, the TPDs cannot depend on absolute time, but only on the time increment. Markov processes whose TPDs have this property, are called homogeneous. It follows that

$$p\left(x^{\left(j\right)},t_{j}|x^{\left(j-1\right)},t_{j-1}\right)=p\left(x^{\left(j\right)},\tau |x^{\left(j-1\right)},0\right),j=1,2,\ldots ,$$ (20)

which holds for any *t_j_*−*t_j_*_−1_= τ≥0.

From Eqs. (19) and (20) it is seen that for a fixed value of the time increment *T*, each of the integrals on the right hand side of Eq. (19) need to be calculated only once, and can be stored for repeated use. That is, the following parameters are calculated initially and stored

$$B_{k_{1}-k_{n}}^{l_{1}-l_{n}}-\int \underset{R^{n}}{•••}\int p\left({x^{\left(j\right)}}_{\left(l_{1}-l_{n}\right),\tau }|x^{\left(j-1\right)},0\right)\overset{n}{\underset{j-1}{⊗}}B_{k_{i}}\left(x^{\left(j-1\right)}\right)dx^{\left(j-1\right)}.$$ (21)

Here, the index *l_i_*, *i*=1,…,*n*, refers to grid point number *l_i_* for the state space variable *x_i_*. It may be noted here that due to the properties of the TPD for small time increments *τ,* the tensor $B_{k_{1}-k_{n}}^{f_{1}-f_{n}}$ has a strongly banded character with the elements decreasing rapidly away from the main diagonal *k*_1_=*l*_1_,…,*k_n_*=*l_n_*. This has important implications for the efficiency of the computer program. Let $p_{l_{1}-l_{n}}^{\left(j\right)}=p\left(x_{\left(l_{1}-l_{n}\right)}^{f},f_{j}|x\prime ,t\prime \right)$. Then Eq. (19) can be rewritten as

$$p_{l_{1}-l_{n}}^{\left(j\right)}=\sum_{k_{l}-1}^{M_{1}}•••\sum_{k_{n}-1}^{M_{n}}\Gamma^{\left(j-1\right)}\left(k1,\ldots ,kn\right)B_{k_{1}-k_{n}}^{l_{1}-l_{n}}.$$ (22)

Having calculated the TPD *p*(*x*^(^*^j^*^)^,*t_j_* | *x*′,*t*′) at the grid points by using Eq. (22), a spline interpolation is again carried out and a new set of interpolation coefficients ${\left\{\Gamma^{\left(l\right)}\left(k_{1},\ldots ,k_{n}\right)\right\}}_{t_{i}-1}^{M_{j}}$ are calculated. This provides an updated representation of the TPD for time step *j*, cf. Eq. (17). For each time step, the normalization condition Eq. (7) is checked. That is, if

$$\begin{array}{c} \int \underset{R^{n}}{•••}\int p\left(x^{\left(j\right)},t_{j}|x\prime ,t\prime \right)dx^{\left(j\right)}=\sum_{k_{l}-1}^{M_{1}}•••\sum_{k_{n}-1}^{M_{n}}1\left(j\right)\left(k_{1},\ldots ,k_{n}\right)\\ \prod_{i-1}^{n}\int_{-\infty }^{\infty }B_{k_{1}}\left(x\right)dx=qj \end{array}$$ (23)

and *q_i_*≠1.0 within the desired accuracy, then the following replacement is made to restore the correct normalization.

$$\Gamma^{\left(j\right)}{\left(k_{1},\ldots ,k_{n}\right)}^{\text{new}}\leftarrow qj^{-1}\Gamma^{\left(j\right)}{\left(k_{1},\ldots ,k_{n}\right)}^{\text{old}}$$ (24)

This normalization check and replacement strategy contributes to producing a very stable and accurate numerical procedure.

## 4. Examples

The accuracy and power of the developed PIS procedure will be illustrated by application to specific case studies taken from two classes of dynamic models. Both models are described by Eq. (3) with *n*−2 and *m*−3. This implies a two-dimensional state space vector *X*=(*X*_1_, *X*_2_)*^τ^*=(*Z,Ż*)*^τ^.* Further, *m*(•) and *Q*(•) are such that *m*_1_(*X*_1_, *X*_2_)=*X*_2_ and q_1_*_j_*(•)=0 for *j*=l,2,3. Assuming sufficient restrictions on *m*(•) and *Q*(•), cf. Refs. [6,7], *X*(*t*) becomes a Markov diffusion process. Invoking Eq. (13), it can be shown that, up to correction terms of order *τ*^2^, the associated TPD assumes the form

$$p\left(x,\tau |x\prime ,0\right)=\delta \left(x_{1}-{x_{1}}^{\prime }-{x_{2}}^{\prime }\tau \right)•\tilde{p}\left(x_{2},\tau |x\prime ,0\right).$$ (25)

$\bar{p}\left(x_{2},\tau |x\prime ,0\right)$ is given by the relation

$$\tilde{p}\left(x_{2},\tau |x\prime ,0\right)-\frac{1}{\sqrt{2\pi \beta \left(x\prime \right)\tau }}\exp\left\{-\frac{{\left(x_{2}-{x_{2}}^{\prime }-m_{2}\left(x\prime \right)\tau \right)}^{2}}{2\beta \left(x\prime \right)\tau }\right\}.$$ (26)

where

$$\beta \left(x\prime \right)=\sum_{j-1}^{3}q_{2_{j}}\left(x\prime \right)2.$$ (27)

By combining Eqs. (25) and (26), and applying the solution technique described in the previous section, the TPD *p*(*x,t | x′,t′*) for large *t−t′* can be calculated. By this, the time evolution of the system when it starts from rest, for example, can be studied. The stationary PDF is obtained in the limit as *t*−*t′*→∞. For application of the PIS method to other problems involving both two- and three-dimensional state space vectors, the reader may consult Reft. [11–13,18,19].

### 4.1 Example 1—The Caughey Oscillator

There is a class of dynamic models for which there exist an analytical solution for the stationary joint PDF of *X.* A member of this class may be called a Caughey oscillator, Ref. [20]. The generic equation of motion for this oscillator can be written as

$$\ddot{Z}+\dot{Z}g\left(E\right)+h\left(Z\right)=\Gamma N\left(t\right).$$ (28)

*N*(*t*) denotes a stationary, zero-mean Gaussian white noise satisfying *E*[*N*(*t*) *N*(*t*+*τ*)]=δ(*τ*), where δ(•) denotes Dirac’s delta function, *Γ* is a positive constant and *g*(*E*) is a function of the total energy *E=E*(*Z,Ż*) given as follows

$$E=\frac{1}{2}{\dot{Z}}^{2}+V\left(Z\right)$$ (29)

where

$$V\left(Z\right)-\int_{0}^{t}h\left(s\right)ds.$$ (30)

For this example *m*_2_(z, ż)= *−*ż*g*[*E*(*z*,ż)]*−h*(*z*), and we may choose *q*_21_*=q*_22_*=W*_1_=*W*_2_=0.* q*_23_=*Γ* and *dW*_3_(*t*)=*N*(*t*)d*t.* The stationary, joint PDF, denoted by p,(•), is then determined by the relation, cf. Refs. [20, 21]

$$p_{s}\left(z,\dot{z}\right)=C\exp\left\{-\frac{2}{\Gamma^{2}}\int_{0}^{E^{\prime }}g\left(s\right)ds\right\},$$ (31)

where *E*′=*ż*^2^/2+*V*(*z*), and *C* is a normalization constant to ensure a total probability equal to 1.0.

For the illustration purposes in this paper, we have chosen the following special case of Eq. (28)

$$\begin{array}{c} \ddot{Z}\left(t\right)+2\xi \dot{Z}\left(t\right)\left\{1+\varepsilon {\left[\frac{1}{2}{\dot{Z}}^{2}\left(t\right)+\frac{1}{2}Z^{2}\left(t\right)+\frac{1}{4}\lambda Z^{4}\left(t\right)\right]}^{1/2}\right\}+Z\left(t\right)\\ +\lambda Z^{3}\left(t\right)=2\sqrt{\xi N\left(t\right)} \end{array}$$ (32)

with parameters ξ, *ε*, and λ.

**Fig. 1:**
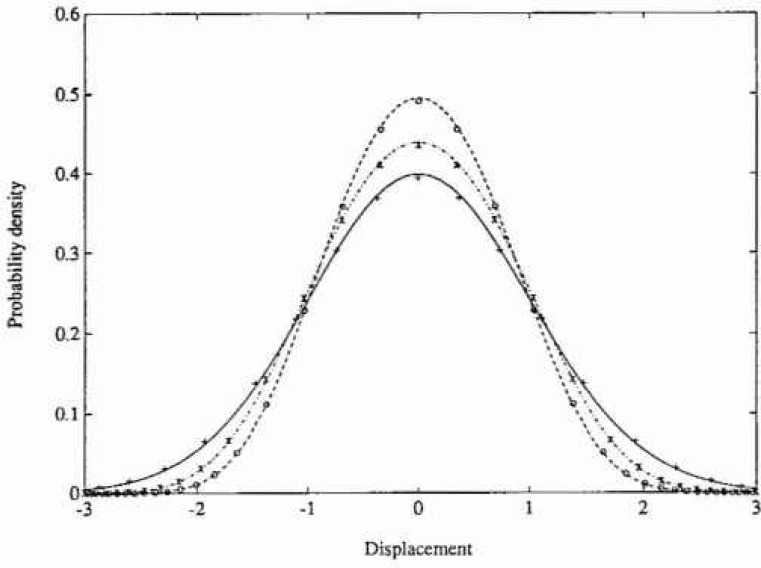
Probability density function of displacement response for the Caughey oscillator in example 1. Analytical solutions: —, *ε*=0, λ=0; -*-*, *ε*=0, λ=0.2;- - - -. *ε*=0.5. λ=0.1, Numerical path integral solution: +, *ε*=0, λ=0; ×, *ε*=0), λ=0.2; O, *ε*=0.5, λ=0.1.

**Fig. 2:**
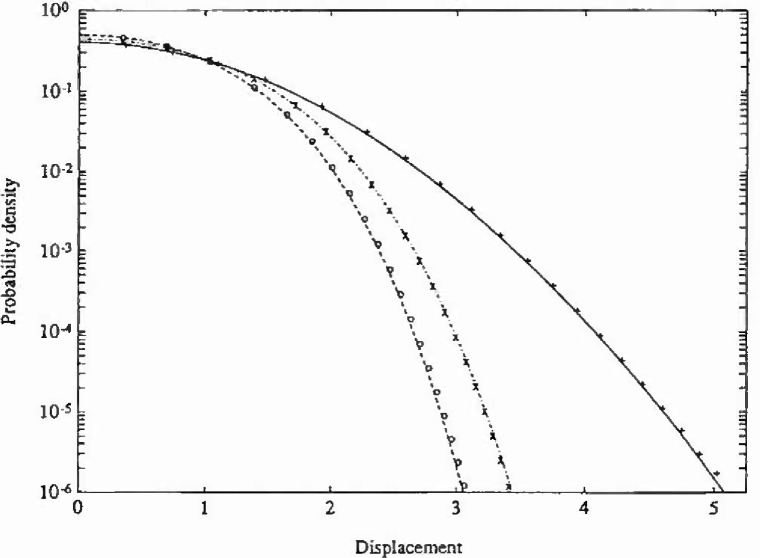
Logarithmic plot of the probability density function of displacement response for the Caughey oscillator in example 1. Key as in Fig. 1.

**Fig. 3:**
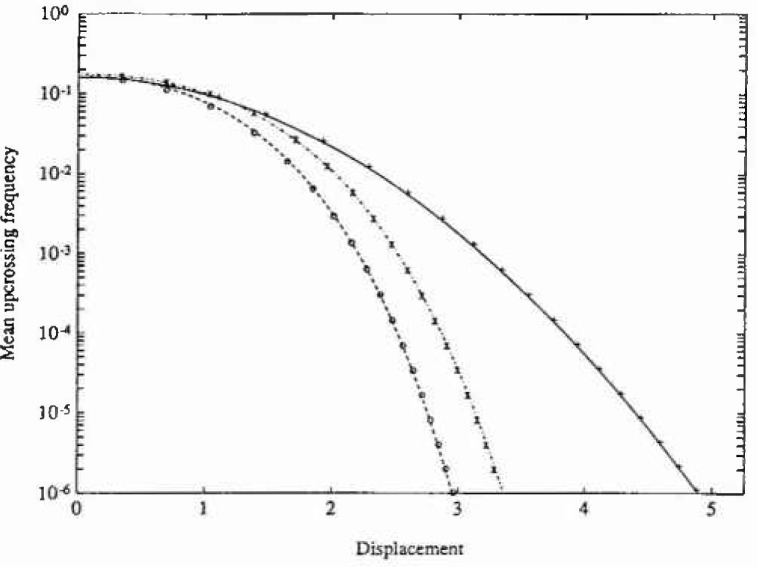
Mean upcrossing rate of displacement response for the Caughey oscillator in example 1. Key as in Fig. 1.

The stationary PDF only depends on the parameters *ε* and λ, and the numerical solution for the following set of parameter values has been calculated (*ε*,λ)=(0,0) (Gaussian response), (0, 0.2) (Duffing oscillator) and (0.5, 0.1). The calculations were carried out with the same number of grid points on both axes in state space, aviz., 45. Since the resulting PDFs are actually independent of *ξ*, the value *ξ*=0.1 was chosen for the Gaussian and Duffing cases, while *ξ*-0.5 was adopted for the last esse. The time increments used were *τ*-00025 s, 0.001 s, and 0.02 s, respectively. The total CPU time on a DEC station 3100^1^ was about 5 minutes for each case. In Figs. 1 and 2 are shown the marginal PDFs of the displacement response for the three case studies considered, together with the corresponding analytical solutions. In Fig. 3 are given the corresponding analytical and numerical results for the mean upcrossing rate. It is seen that in all three cases the agreement between the numerical PIS and the analytical solution is very good over the whole range of probability levels given. In fact, the accuracy can be retained down to much lower probability lewis (=10^−l0^) at a moderate increase in computer time.

### 4.2 Example 2—Parametric and External Excitation

In this example, the response statistics of a nonlinear oscillator subjected to both external and parametric random excitation will be illustrated by applying the methodology of the paper to two specific case studies.

The equation of motion of the oscillator is the following

$$\ddot{Z}+2\xi \left[1+{\tilde{N}}_{1}\left(t\right)\right]\dot{Z}+\gamma \left[Z^{2}+\frac{Z^{2}}{\omega_{0}^{2}}\right]\dot{Z}+\omega_{0}^{2}\left[1+{\tilde{N}}_{2}\left(t\right)\right]Z-{\tilde{N}}_{3}\left(t\right).$$ (33)

Here ξ, *γ*, and *ω*_0_ are positive constants, *Ñ*,(*t*), *j*=1,2,3, are independent Gaussian white noises satisfying

$$E\left[{\tilde{N}}_{j}\left(t\right){\tilde{N}}_{j}\left(t+\tau \right)\right]=\Gamma_{j}^{2}\delta \left(\tau \right).j=1.2.3.$$ (34)

where *Γ_j_* are positive constants. For this example it is found that $m_{2}\left(Z,\dot{Z}\right)=-2\xi \dot{z}-\gamma \left[z^{2}+{\dot{z}}^{2}/\omega_{\upsilon }^{2}\right]-\omega_{\upsilon }^{2}z,$ $q_{21}\left(Z,\dot{Z}\right)=-2\xi \dot{z}\Gamma_{1},q_{22}\left(z,\dot{z}\right)=-\omega_{\upsilon }^{2}z\Gamma_{2}$ and $q_{23}\left(z,\dot{z}\right)=\Gamma_{3}$.

This model was studied by Dimentberg [22], who showed that when

$$\omega_{0}^{2}\Gamma_{3}^{2}=4\xi^{2}\Gamma_{1}^{2}$$ (35)

a closed-form expression for the stationary joint PDF can be obtained. It is given as

$$p_{5}\left(z,\dot{z}\right)=C\frac{\exp\left\{-\mu \left(z^{2}+{\dot{z}}^{2}/\omega_{0}^{2}\right)\right\}}{{\left(\kappa +z^{2}+{\dot{z}}^{2}/\omega_{0}^{2}\right)}^{\delta -\kappa \mu }},$$ (36)

where C is a normalization constant and

$$\kappa =\frac{\Gamma_{3}^{2}}{\omega_{0}^{4}\Gamma_{2}^{2}},\delta =\frac{2\xi }{\omega_{0}^{2}\Gamma_{2}^{2}}+\frac{1}{2},\mu =\frac{\gamma }{\omega_{0}^{2}\Gamma_{2}^{2}}$$ (37)

By this, we have the opportunity to test the accuracy of the PIS method for this kind of dynamic model. The results of two particular cases will be presented.

**Fig. 4:**
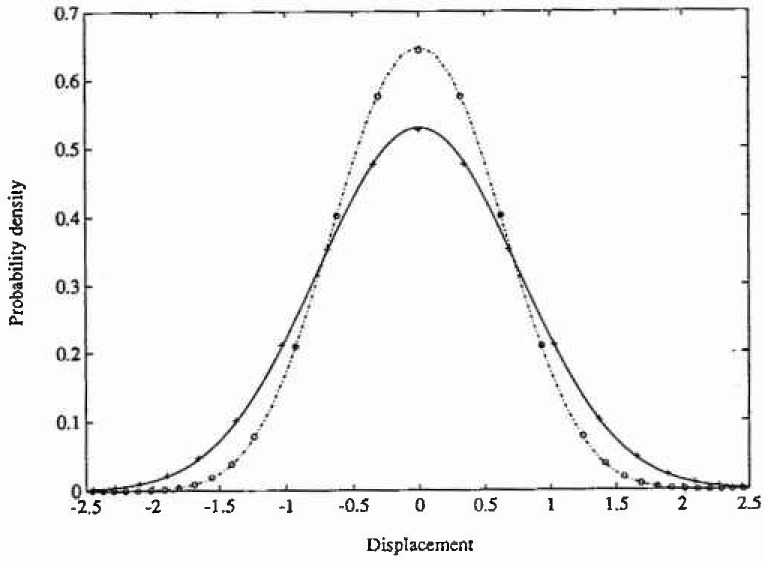
Probability density function of displacement response for the oscillator in example 2, case 1 and 2. Analytical solutions: —. case 1; -•-•, case 2. Numerical path integral solution: +, case 1; O. case 2.

**Fig. 5:**
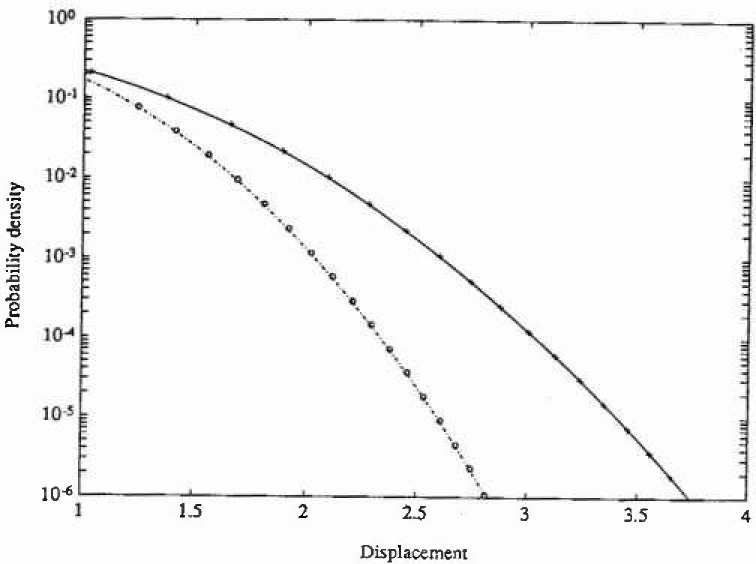
Logarithmic plot of the probability density function of displacement response for the oscillator in example 2, case 1 and 2, Key as in Fig. 4.

**Fig. 6:**
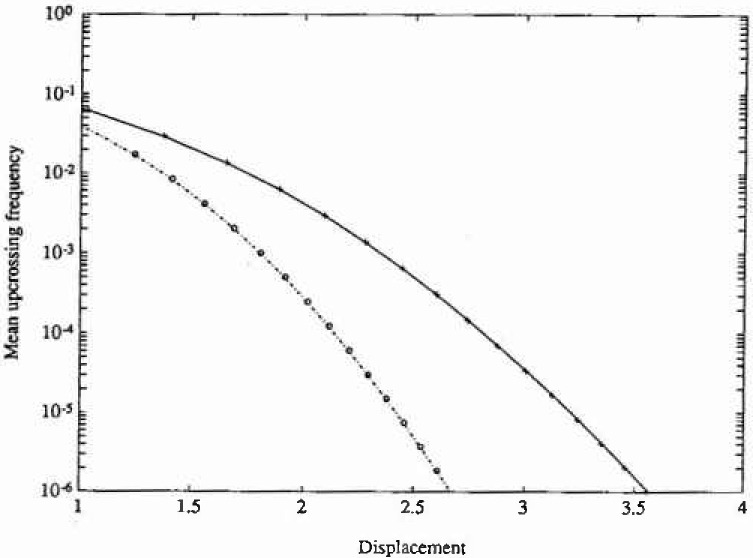
Mean upcrossing rate of displacement response for the oscillator in example 2. case 1 and 2. Key as in Fig. 4.

**Case 1:** Here the following parameter values were used. $\xi =0.1,\gamma =0.1,\omega_{0}=1.0,\Gamma_{1}^{2}=2.5,\Gamma_{2}^{2}=0.1,\Gamma_{3}^{2}=0.3$. For the numerical calculations a grid size of 49×49 points and a time increment *τ*=0.01 s was used. The total CPU time on a DEC 3100 work station was 3 min for the PIS calculation. The results for the analytical and numerical solutions are given in Figs. 4–6. In Figs. 4 and 5 are shown the marginal PDF of the displacement response and in Fig. 6 is shown the corresponding mean upcrossing rate.

**Case 2:** In this case the following set of parameters were used. $\xi =0.1,\gamma =0.4,\omega_{0}=1.0,\Gamma_{1}^{2}=5.0,\Gamma_{2}^{2}=0.2,\Gamma_{3}^{2}=0.3$. A grid size of 51×51 points together with a time increment *τ* = 0.01 S were chosen. The CPU time was about the same as in the previous case. The same results as for Case 1 are presented in Figs. 4–6.

## 5. Conclusions

A numerical method for estimating the extreme response of nonlinear oscillators excited by white noise, or filtered white noise, has been described. The example calculations presented show that the method gives very accurate estimates of the required joint PDF. In fact, for every example having analytical solution on which the method has been tested, complete agreement has been found with proper choice of grid size and time increment in the numerical solution procedure. In the present paper, of course, only a few cases can be given. Experience with the method indicates that two-dimensional problems can be solved routinely with high accuracy requiring a few minutes CPU time on a work station (DEC station 3100). The solution of three-dimensional problems requires more care in the sense that computer capacity starts to become an issue of importance. In such cases the CPU time easily runs into hours.

## Footnotes

1 Certain commercial equipment, instruments, or materials are identified in this paper to specify adequately the experimental procedure. Such identification does not imply recommendation or endorsement by the National Institute of Standards and Technology, nor does it imply that the materials or equipment identified are necessarily the best available for the purpose.
